# Ex Vivo Analysis of the Antimicrobial Effect of Diode Laser and Sodium Hypochlorite on *Enterococcus Faecalis* Atcc 29212

**DOI:** 10.34172/joddd.43918

**Published:** 2026-06-30

**Authors:** Germania Patricia Torres Veintimilla, Mauricio Aguirre Balseca, Karina Maria Salvatore Freitas, Boris Hernán Villacrés Granda

**Affiliations:** ^1^Department of Health Sciences, University of the Hemispheres, Quito, Ecuador; ^2^Department of Orthodontics, Ingá University Center UNINGA, Maringá, Brazil, Bauru Dental School, University of São Paulo, Bauru, Brazil; ^3^Department of Health Sciences, University of the Hemispheres, Quito, Ecuador

**Keywords:** Disinfection, Enterococcus faecalis, Laser, PDT, Sodium hypochlorite

## Abstract

**Introduction:**

The present study evaluated the efficacy of the diode laser in eliminating *Enterococcus faecalis* ATCC 29212 from root canals through ex vivo experiments.

**Methods:**

Ninety single-rooted premolars were prepared with Endogal files, incubated anaerobically at 37ºC for 14 days, and randomly assigned to six groups (n=15 per group): G1: irrigation with 5.25% sodium hypochlorite (NaOCl); G2: NaOCl plus diode laser L1 (660 nm, 100 mW, 90 s); G3: NaOCl plus diode laser L2 (808 nm, 100 mW, 90 s); G4: L1 alone; G5: L2 alone; and G6: 0.9% sodium chloride (NaCl) as control. Root canal samples were collected using sterile paper points, and bacterial load was assessed by mass plating on Mueller‒Hinton agar, followed by incubation at 37ºC for 24 hours to quantify colony-forming units (CFU/mL).

**Results:**

The combination of NaOCl and photodynamic therapy (PDT) (G2) significantly reduced bacterial counts compared with NaOCl alone (G1) (Z=2.89, adjusted *P*=0.01), and NaOCl combined with the 808 nm laser (G3) was significantly more effective than PDT alone (G4) (adjusted *P*<0.001). When applied without NaOCl, both laser-based protocols were significantly less effective than NaOCl alone: NaOCl (G1) produced significantly greater bacterial reduction than PDT alone (G4) (Z=−2.50, adjusted *P*=0.04), followed by the 808 nm laser alone (G5) (Z=−4.40, adjusted *P*<0.001).

**Conclusion:**

The combination of 5.25% NaOCl with PDT produced the lowest *E. faecalis* counts and was significantly more effective than NaOCl alone, followed by the laser protocols applied without NaOCl. However, it did not differ significantly from NaOCl combined with the 808 nm laser, indicating that its advantage over other NaOCl-based protocols was numerical rather than statistically confirmed.

## Introduction

 The disinfection of the root canal system aims to eliminate microorganisms that form biofilms on the canal walls, along with residual pulp tissue, thereby creating a favorable environment for periapical tissue healing.^1^ Microbial control is crucial during endodontic procedures, including chemomechanical preparation, obturation, and sodium hypochlorite (NaOCl) irrigation. These steps play a key role in disrupting the biofilm and reducing bacterial load.^2^

 However, the complex root canal anatomy can reduce the effectiveness of NaOCl.^3^ This challenge has driven the exploration of adjunctive methods, such as photodynamic therapy (PDT).^4,5^ Chemomechanical preparation is fundamental for minimizing bacteria colonizing the dentinal tubules.^6^ Among various disinfectants, NaOCl is the most widely used due to its proteolytic and tissue-dissolving properties, particularly against opportunistic pathogens like *E. faecalis*.^7^

*E. faecalis* is a Gram-positive, facultative anaerobe often found in periapical lesions.^8^ It survives within dentinal tubules due to its virulence mechanisms, notably adhesion to dentinal collagen via biofilm formation.^5^ Its lipoteichoic acid contributes to chronic inflammation, persisting within root canals even after bacterial death.^9^

 Rotary instruments leave significant portions of root canal walls untouched, allowing bacterial penetration up to 1150 μm into dentinal tubules, whereas chemical irrigants penetrate only about 100 μm. *E. faecalis* can penetrate even deeper, up to 1000 μm within three weeks.^1^

 Laser technology has been proposed to enhance root canal disinfection by improving the outcomes of conventional irrigation. Lasers emit concentrated energy, leading to varied tissue effects depending on wavelength, power, and exposure time.^7^ Er: YAG lasers operate via shock waves and fluid dynamics induced by rapid bubble expansion and collapse.^10^ Diode lasers, classified by power, range from low-level (632–830 nm, 1–100 mW) to high-power devices (810–980 nm, 1–15 W).^11^

 Particularly, the 980 nm diode laser achieves deep tissue penetration, with high absorption in melanin and hemoglobin, while sparing water and hydroxyapatite, thus producing effective thermal and hemostatic effects compared to Er: YAG laser.^2,7^

 Near-infrared diode lasers (810–980 nm) show significant antibacterial photothermal effects, efficiently reducing bacteria deep within dentinal tubules.^9^ Flexible optical fibers (200–320 μm) facilitate root canal access but are limited by axial light emission, reducing lateral efficacy.^12^ The DUO diode laser (MMO) offers two modes: L1 (660 nm with methylene blue (MB) at 0.005%) and L2 (808 nm), both delivering 100 mW for 90 seconds.

 The two wavelengths investigated in this study were selected because they represent distinct antimicrobial mechanisms and clinically relevant adjunctive approaches in endodontic disinfection. The 660 nm wavelength falls within the red light spectrum and closely matches the absorption peak of MB, making it particularly suitable for PDT by generating reactive oxygen species.^13,14^ In contrast, the 808 nm wavelength is in the near-infrared spectrum and has been associated with a predominantly photothermal antibacterial effect, enabling deeper penetration into dentinal tubules and enhanced microbial reduction.^9^ Evaluating these wavelengths under standardized conditions enabled the comparison of two different disinfection strategies: a photochemical approach, combining 660 nm irradiation with MB, and a photothermal approach, using 808 nm laser irradiation alone. Both strategies have previously demonstrated potential as adjunctive methods to conventional root canal disinfection protocols.^1,14^

 Nonetheless, laser use poses a risk of heat transfer to external root surfaces, potentially damaging the alveolar bone depending on heat generation and duration.^12,15^

 PDT complements conventional root canal disinfection,^8^ employing light-activated photosensitizers such as MB to generate reactive oxygen species, thereby causing microbial death.^16,17^ MB absorbs light around 670 nm and is optimally activated by red light between 630–700 nm.^18^ However, excessive power density may deplete oxygen in poorly perfused tubules, impairing bacterial elimination.^13^ An MB concentration between 0.001% and 0.01% is ideal to effectively inhibit *E. faecalis*.^16^

 Therefore, this study aims to evaluate the antimicrobial efficacy of the DUO diode laser at different wavelengths (660 nm and 808 nm) with 100 mW output, alongside 5.25% NaOCl, against *E. faecalis* in ex vivo endodontic treatment.

## Methods

 This study was approved by the Ethics Committee of the University of the Hemispheres, Quito, Ecuador (protocol n. CEUHE25-47).

###  Experimental Design

 An ex vivo, experimental, quantitative, and descriptive study was conducted at the Microbiology Laboratory of the University of the Hemispheres, Quito, Ecuador, which complies with biosafety standards for the cultivation, inoculation, and susceptibility testing of the bacterial strain under study.

 Ninety single-rooted premolars were selected, and the experimental unit was defined as the individual tooth/root canal. The specimens were randomly allocated to six groups (n = 15 per group) using a computer-generated random sequence, performed by an investigator not involved in the disinfection procedures. Because the disinfection protocols differ in their mode of application, the operator performing the treatments could not be blinded to group allocation; however, the samples were coded, and the microbiologist who counted the CFU and the investigator who performed the statistical analysis were blinded to group allocation.

 The experimental unit was defined as the individual tooth/root canal. The groups were distributed according to the disinfection protocol as follows:

 G1: irrigated with 5 mL of 5.25% NaOCl for 5 cycles of 20 seconds

 G2: irrigated with 5 mL of 5.25% NaOCl for 5 cycles of 20 seconds, then 40 μL of 0.005% MB was applied as a photosensitizer and irradiated with the Duo MMO laser configured at L1 (wavelength of 660 nm, output power of 100 mW) for 90 seconds using an optical fiber

 G3: irrigated with 5 mL of 5.25% NaOCl for 5 cycles of 20 seconds, then irradiated with the diode laser configured at L2 (wavelength of 808 nm, output power of 100 mW) for 90 seconds

 G4: 0.005% MB was applied up to the cavity level using a disposable syringe; this solution was agitated with a K#15 file for 2 minutes within the root canal, then irradiated with the diode laser configured at L1 for 90 seconds

 G5: diode laser configured at L2 applied for 90 seconds

 G6: control group irrigated with 5 mL of 0.9% NaCl for 5 cycles of 20 seconds

###  Sample Preparation

 The 90 selected single-rooted premolars (population) were stored in 0.9% NaCl and kept refrigerated at 4ºC until use. To ensure a sterile environment, the samples were autoclaved at 120ºC for 15 minutes at 1 atmosphere. To confirm sterility, the teeth were placed in brain-heart infusion (BHI) broth and incubated for 24 hours at 37ºC under anaerobic conditions. Subsequently, 100 µL of the sample was plated onto plate count agar and incubated again at 37ºC for 24 hours under anaerobic conditions. The absence of bacterial colonies was confirmed.

 For the application of different treatments, each dental organ was sectioned at the cementoenamel junction using a cylindrical diamond bur, standardizing a working length of 15 mm. All root canals were instrumented with the Endogal file sequence. Root canal patency was achieved using the manual Pre K12.01 file, while rotary instrumentation was performed following the manufacturer’s instructions at 350 rpm with a torque of 4 N, using files A (Ø = 0.15 mm), B (Ø = 0.20 mm), D (Ø = 0.25 mm), and E (Ø = 0.30 mm) as the master apical file. For proper disinfection, each root canal was irrigated with 1 mL of 5.25% NaOCl between each instrument, using a 5-mL disposable syringe with a side-vented needle.

###  Characterization of the Microbial Strain E. faecalis ATCC 29212

 The pathogenic microorganism *E. faecalis* ATCC 29212 was obtained from the General Microbiology Laboratories at the University of the Hemispheres in Quito, Ecuador. The strain was activated by inoculating it onto a Petri dish containing nutrient agar, which was then incubated at 37ºC for 24 hours. Bacterial colonies were observed and characterized by Gram staining, confirming their Gram-positive coccus status.

 To determine the genus, biochemical tests were performed: catalase, hemolysis, 6.5% NaCl tolerance, blood agar, bile esculin, and motility. For species identification, specific tests were used: arabinose acidification (phenol red broth, arabinose 5 g/L, pH = 7.3 ± 0.2) and tellurite tolerance (trypticase soy agar with 0.04% potassium tellurite, pH = 7.3 ± 0.2).

###  Inoculation of Teeth with E. faecalis 

 The pathogenic microorganism *E. faecalis* was maintained throughout the experiment in BHI broth and plate count agar. To contaminate the teeth, *E. faecalis* was first inoculated onto BHI agar under anaerobic conditions and incubated at 37°C for 24 hours. Subsequently, serial dilutions were performed until obtaining a standard McFarland 0.5 concentration (1.5 × 10⁸ CFU/mL) in 10 mL of BHI broth.

 After the sterility of the specimens was confirmed, each tooth was placed in a test tube containing 10 mL of BHI broth inoculated with *E. faecalis* ATCC 29212, adjusted to a 0.5 McFarland standard (1.5 × 10⁸ CFU/mL), and incubated at 37ºC under anaerobic conditions for 14 days to allow mature biofilm formation. To maintain nutrient availability and bacterial viability, the culture medium was renewed at each reinoculation, and the specimens were reinoculated on days 1, 4, 7, 10, and 14, each time using a freshly prepared *E. faecalis* suspension adjusted to the same 0.5 McFarland standard (1.5 × 10⁸ CFU/mL). Throughout the incubation period, sterile non-inoculated specimens were maintained under the same conditions as negative controls to monitor for contamination, and the presence of *E. faecalis* was confirmed by turbidity of the BHI broth.

 At the end of the incubation period, a gentle rinse with 1 mL of NaCl solution was performed to remove non-adherent bacteria from the external surface of the root, preserving the intact bacterial biofilms formed on the internal walls of the teeth.

###  Disinfection Protocols for Root Canals Contaminated with E. faecalis 

 Prior to applying the disinfection protocols, each test tube containing 10 mL of BHI broth was vortexed for 30 seconds. Then the dental organ was extracted with curved mosquito forceps. Subsequently, the external surface of the root was rinsed with 0.9% NaCl using a 5-mL disposable syringe.

###  Treatment of Root Canals Experimentally Contaminated with E. faecalis 

 Disinfection was performed according to the experimental design (Table 1).

**Table 1:** Experimental design for root canal disinfection against *E. faecalis* ATCC 29212 biofilms (n = 15)

**Groups**	**Disinfection protocol**
Group 1	5.25% NaOCl
Group 2	5.25% NaOCl and L1
Group 3	5.25% NaOCl and L2
Group 4	L1. Wavelength of 660 nm
Group 5	L2. Wavelength of 808 nm
Group 6	0.9% saline solution

Note. L1: Wavelength of 660 nm, with an output power of 100 mW L2: Wavelength of 808 nm, with an output power of 100 mW

 A diode laser (DUO, MMO, MMOptics, São Carlos, Brazil) was operated in continuous-wave mode at two wavelengths: L1 (660 nm) for photodynamic therapy and L2 (808 nm) for direct irradiation, both at an output power of 100 mW for 90 s. The energy was delivered through a 200-µm flexible optical fiber introduced into the root canal and moved continuously in an apicocoronal direction (back-and-forth motion), in contact with the root canal walls. Under these parameters, the total energy delivered per application was 9 J (0.1 W × 90 s), corresponding to a power density of 318 W/cm^2^ and a fluence of 2.87 × 10⁴ J/cm^2^, calculated from the fiber tip area. The laser output was calibrated before each session using a power meter. For the photodynamic therapy groups (G2 and G4), 40 µL of 0.005% methylene blue was used as the photosensitizer; it was introduced into the root canal and left in place for a 2-min pre-irradiation period (in G4, agitated with a size 15 K-file), and the canal was not rinsed after photosensitizer application before irradiation.

 In group 1 (n = 15), the samples were treated exclusively with 5 mL of 5.25% NaOCl for 5 cycles of 20 seconds, using a side-vented needle positioned 1 mm from the working length.

 In group 2 (n = 15), the samples were first irrigated with 5 mL of 5.25% NaOCl for 5 cycles of 20 seconds. Then, for PDT, 40 μL of 0.005% MB was used as a photosensitizer and applied to the root canal for 2 minutes. Using the optical fiber of the Duo MMO laser configured at L1, back-and-forth movements (from apical to incisal) were performed for 90 seconds.

 In group 3 (n = 15), treatment involved 5 mL of 5.25% NaOCl for 5 cycles of 20 seconds, followed by application of the Duo MMO diode laser with a conventional tip configured at L2 for 90 seconds.

 In group 4 (n = 15), 40 μL of 0.005% MB was used as a photosensitizer, applied for 2 minutes while performing back-and-forth movements with the optical fiber of the Duo MMO laser, configured at L1 for 90 seconds.

 In group 5 (n = 15), each root canal was treated with the Duo MMO laser using the conventional tip, configured at L2, for 90 seconds.

 Group 6 (n = 15) served as the negative control, and the root canals were irrigated with 5 mL of 0.9% NaCl for 5 cycles of 20 seconds each.

###  Analysis of the Antimicrobial Effect of the DUO MMO Diode Laser on E. faecalis

 Before microbiological sampling, residual NaOCl was neutralized by irrigating the root canals with sodium thiosulfate solution (concentration: 5%) for 60 seconds to prevent carryover antimicrobial activity during sample collection, incubation, and CFU quantification.

 After completing the disinfection treatments, the presence or absence of *E. faecalis* was verified in each of the 90 teeth, using sterile #25/06 paper points. For this, a paper point was inserted into the root canal and rotated twice clockwise for 20 seconds to collect the sample.

 The sample was then placed in test tubes containing 10 mL of BHI broth, vortexed for 30 seconds, and incubated at 37ºC for 24 hours under anaerobic conditions.

 Subsequently, serial dilutions were prepared up to 1:100,000, and 100 μL of each dilution was plated on Petri dishes with plate count agar, followed by incubation at 37ºC for 24 hours under anaerobic conditions.

 Finally, the number of colony-forming units (CFU/mL) was counted.

###  Statistical Analysis

 Statistical analysis was performed using R statistical software. Normality tests were applied to assess data distribution. Since the data did not follow a normal distribution, CFU/mL values were analyzed using the Kruskal–Wallis test, followed by Dunn’s post hoc test with Holm adjustment for pairwise comparisons. A significance level of α = 0.05 was adopted.

 The main comparison of interest was between 5.25% NaOCl combined with photodynamic therapy (G2) and 5.25% NaOCl alone (G1), as it directly addressed whether photodynamic therapy provided additional antibacterial benefits beyond conventional irrigation. The remaining pairwise comparisons were considered exploratory.

 A post hoc power analysis was performed based on the results of the Kruskal–Wallis test. The observed effect size was estimated using epsilon-squared (ε^2^), calculated from the H statistic, total sample size, and number of groups.

## Results

Table 2 presents the descriptive statistics of the experimental groups. The Kruskal–Wallis test revealed significant differences in bacterial colony counts between the disinfection protocols (H = 66.14, *P* < 0.001) (Table 3).

**Table 2:** Descriptive statistics of colony-forming unit counts per milliliter (CFU/mL) following the treatments used in the disinfection of root canals inoculated with *E. faecalis* ATCC 29212.

**Group**	**Min.**	**1.Qu** **CFU/mL**	**Median** **CFU/mL**	**Mean** **CFU/mL**	**3.Qu** **CFU/mL**	**Max.** **CFU/mL**
G1: NaOCl 5.25 %	2,00 x 10^3^	2,00 x 10^3^	3,00 x 10^3^	3,00 x 10^3^	4,00 x 10^3^	5,00 x 10^3^
G2: NaOCl 5.25 % + L1	0,00	0,00	0,00	5,30 x 10^2^	1,00 x 10^3^	2,00 x 10^3^
G3: NaOCl 5.25 % + L2	0,00	1,50 x 10^3^	2,00 x 10^3^	2,10 x 10^3^	3,00 x 10^3^	4,00 x 10^3^
G4: L1	2,00 x 10^5^	2,04 x 10^5^	2,06 x 10^5^	2,06 x 10^5^	2,08 x 10^5^	2,20 x 10^5^
G5: L2	4,50 x 10^5^	5,03 x 10^5^	5,08 x 10^5^	5,06 x 10^5^	5,11 x 10^5^	5,26 x 10^5^

Note. L1: Wavelength of 660 nm, with an output power of 100 mW. L2: Wavelength of 808 nm, with an output power of 100 mW.

**Table 3:** Kruskal-Wallis test to determine significant differences in the number of bacterial colonies per milliliter (CFU/mL) among the groups analyzed for *E. faecalis* ATCC 29212

**Variable**	**Test**	**H-statistic**	***P*** **-value**
Treatment	Kruskal-Wallis	66.14	< 0.001

 Pairwise comparisons were subsequently performed using Dunn’s post hoc test with Holm adjustment. The main comparison of interest was between NaOCl alone (G1) and NaOCl combined with photodynamic therapy (G2). The combination of NaOCl and PDT (G2) resulted in a significantly greater reduction in bacterial colonies than NaOCl alone (G1) (Z = 2.89, adjusted P = 0.01). No significant difference was observed between NaOCl alone (G1) and NaOCl combined with L2 (G3) (Z = 0.94, adjusted *P* = 0.34).

 NaOCl alone (G1) significantly reduced bacterial colonies compared with PDT alone (G4) (Z = -2.50, adjusted *P* = 0.04) and L2 alone (G5) (Z = -4.40, adjusted *P* < 0.001). No significant difference was observed between NaOCl combined with PDT (G2) and NaOCl combined with L2 (G3) (Z = -1.94, adjusted *P* = 0.15).

 Both NaOCl combined with PDT (G2) and NaOCl combined with L2 (G3) showed significantly lower bacterial counts than PDF alone (G4) and L2 alone (G5) (all adjusted *P* < 0.001). No significant difference was observed between PDT alone (G4) and L2 alone (G5) (Z = -1.89, adjusted *P* = 0.11) (Table 4) (Figure 1).

**Table 4:** Pairwise comparisons performed using Dunn’s post hoc test with Holm-adjusted *P* values to compare the antibacterial efficacy of treatments in the disinfection of root canals inoculated with *E. faecalis*

**Protocol comparison**	***P*** ** – value**	**Level of significance**	**Z**
NaOCl 5.25 % (G1) x NaOCl 5.25 % + L1 (G2)	0.01	0.05	2.89
NaOCl 5.25 % (G1) x NaOCl 5.25 % + L2 (G3)	0.34	0.05	0.94
NaOCl 5.25 % (G1) x L1 (G4)	0.04	0.05	-2.50
NaOCl 5.25 % (G1) x L2 (G5)	< 0.001	0.05	-4.40
NaOCl 5.25 % + L1 (G2) x NaOCl 5.25 % + L2 (G3)	0.15	0.05	-1.94
NaOCl 5.25 % + L1 (G2) x L1 (G4)	< 0.001	0.05	-5.40
NaOCl 5.25 % + L1 (G2) x L2 (G5)	< 0.001	0.05	-7.29
NaOCl 5.25 % + L2 (G3) x L1 (G4)	< 0.001	0.05	-3.45
NaOCl 5.25 % + L2 (G3) x L2 (G5)	< 0.001	0.05	-5.35
L1 (G4) x L2 (G5)	0.11	0.05	-1.89

Note. L1: Wavelength of 660 nm, with an output power of 100 mW. L2: Wavelength of 808 nm, with an output power of 100 mW

**Figure 1:**
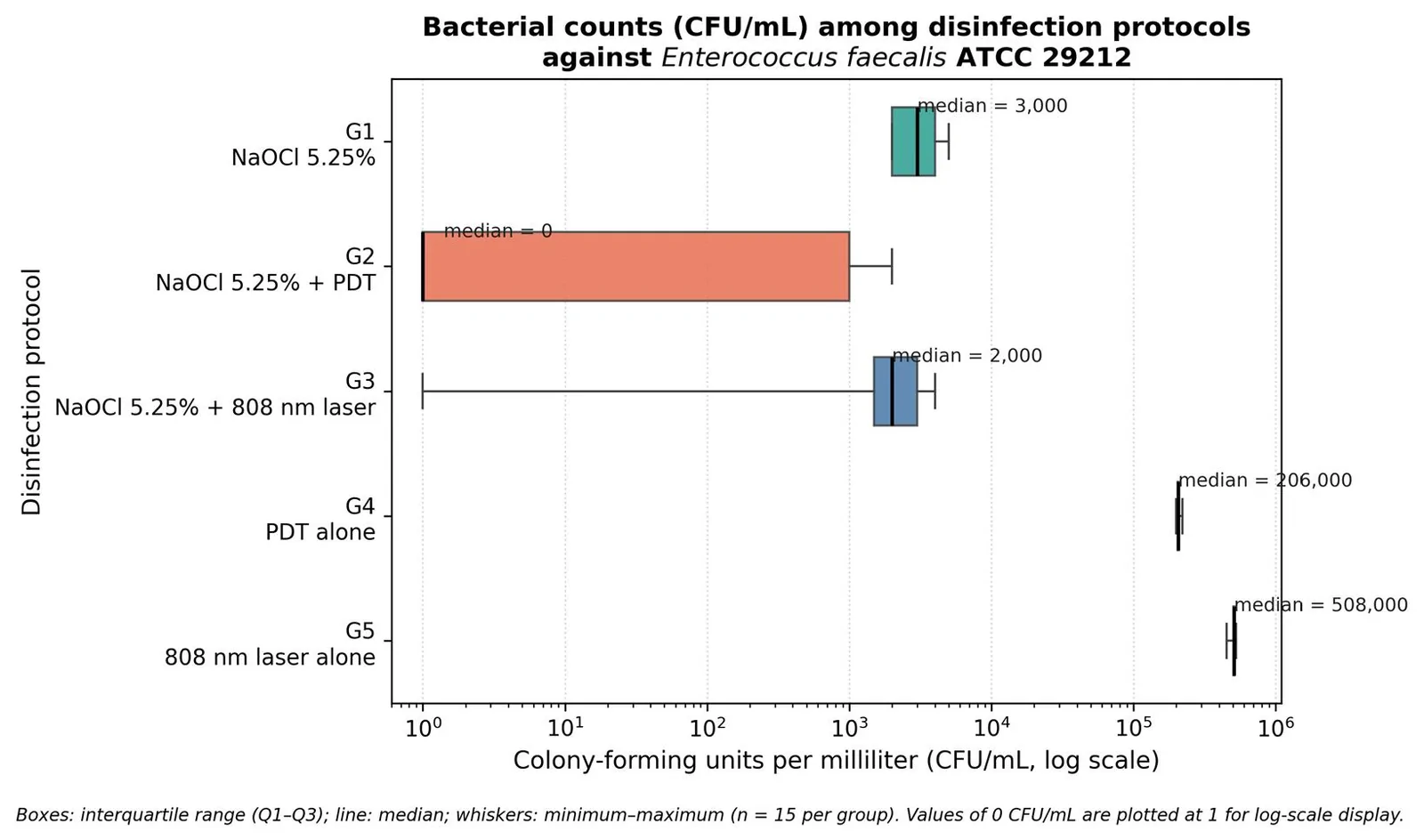
Colony-forming unit counts (CFU/mL) after each disinfection protocol against *E. faecalis*. G1: 5.25% NaOCl; G2: 5.25% NaOCl + PDT (660 nm diode laser + 0.005% MB); G3: 5.25% NaOCl + 808 nm diode laser; G4: PDT alone; G5: 808 nm diode laser alone. Boxes represent the interquartile range (Q1–Q3), the central line indicates the median, and the whiskers represent the minimum and maximum values (n=15 per group). The x-axis is on a logarithmic scale; values of 0 CFU/mL are plotted at 1 to maintain a logarithmic display

 The control group showed the highest bacterial counts, serving as a descriptive reference for bacterial growth in the absence of active disinfection protocols.

## Discussion

 Several studies have demonstrated that the goal of root canal disinfection protocols is to eliminate or reduce microorganisms within the main canal, thereby facilitating the repair of periapical tissues.^16^ Beltes et al.^19^ evaluated the antimicrobial efficacy against *E. faecalis* using PDT with indocyanine green. The results indicated that the combination of 2.5% NaOCl and PDT achieved a significantly greater reduction in CFU/mL than either 2.5% NaOCl or PDT alone.^19^ These findings are consistent with our study, where the combination of 5.25% NaOCl and PDT resulted in the greatest reduction of bacterial colonies.

 Despite this, chemomechanical preparation with NaOCl, at different concentrations, does not fully eliminate bacteria present in accessory canals and isthmuses. Approximately 40‒60% of microbiological analyses report the persistence of apical periodontitis following endodontic treatment.^20^

 Masuda et al.^21^ examined the bactericidal effectiveness of PDT, 2.5% NaOCl, and 2% chlorhexidine against *E. faecalis*. The results showed a notable reduction in bacterial load (CFU/mL) that was statistically significant across all evaluated groups. Interestingly, the combination of 2.5% NaOCl and PDT with a diode laser completely eliminated bacterial colonies, compared to either PDT, NaOCl, or chlorhexidine treatments alone.^22^ These results are consistent with the findings reported in the present study, where a similar reduction of bacterial colonies was observed.

 Romeo et al.^7^ employed KTP (potassium titanyl phosphate) and 980 nm diode lasers to reduce *E. faecalis* in root canals. When mechanical instrumentation, irrigation with 5% NaOCl, and laser irradiation were combined, a considerable reduction in CFU/mL was observed compared with 5% NaOCl alone. In that study, laser irradiation alone did not produce a significantly greater reduction in bacterial load than 5.25% NaOCl. This is partly consistent with our findings, in which NaOCl combined with the 808 nm laser (G3) did not differ significantly from NaOCl alone (G1); however, in our study, the laser protocols applied without NaOCl (G4 and G5) were significantly less effective than NaOCl alone.

 Borges et al.^1^ evaluated the antibacterial effect of the diode laser at 808 nm and 970 nm wavelengths on the decontamination of infected root dentin. The results indicated that treatment with 2.5% NaOCl produced a significant reduction in pathogen proliferation, achieving results comparable to those obtained with groups treated with 2.5% NaOCl combined with the diode laser at 808 nm and 970 nm, respectively. However, our results differ from the above findings, as no statistically significant difference was observed between NaOCl alone (G1) and NaOCl combined with the 808 nm laser (G3) (adjusted *P* = 0.34).

 Conversely, using the diode laser followed by conventional irrigation with 2.5% NaOCl did not yield a significant reduction in bacterial load. These results differ from those obtained in our research, possibly due to differences in the laser wavelength, output power, bacterial strain used, and the protocols applied.^1^

 Moritz et al.^11^ analyzed the antimicrobial effectiveness of the diode laser against Gram-positive and Gram-negative bacteria. A significant reduction in *E. faecalis* was observed after the second application of an 808 nm diode laser at 2 W, with complete eradication of the pathogenic bacteria after the fifth irradiation. The results obtained in our research contrast with these findings, which may be attributed to differences in the number of laser applications, the bacterial strain used, and the protocol followed. In our study, only one application of L2 was performed, which could explain the differences in the results.^11^

 Although no significant difference was found between the two laser protocols applied without NaOCl (PDT alone in G4 versus the 808 nm laser alone in G5; adjusted *P* = 0.11), the combination of 5.25% NaOCl with PDT (G2) produced the lowest CFU/mL counts and was significantly more effective than NaOCl alone (G1), followed by the laser protocols applied without NaOCl (G4 and G5). It was not, however, significantly different from NaOCl combined with the 808 nm laser (G3) (adjusted *P* = 0.15). Therefore, although NaOCl–PDT ranked highest numerically, it can be regarded as the most effective protocol only relative to NaOCl alone and to laser-only approaches; its advantage over NaOCl combined with the 808 nm laser was not statistically confirmed.

 From a clinical perspective, the implementation of PDT and diode lasers should also be evaluated for cost-effectiveness and practical feasibility. Although these technologies may enhance root canal disinfection when used as adjunctive procedures, their incorporation into routine clinical practice requires additional equipment, operator training, and increased chairside time. Consequently, their use may be more justifiable in complex cases, persistent infections, or retreatment procedures in which conventional chemomechanical preparation alone may be insufficient. Future clinical studies should investigate not only their antimicrobial efficacy but also their cost-benefit ratio and their impact on treatment efficiency and long-term outcomes.

 A further consideration is the thermal effect associated with diode laser irradiation. In the present study, external root surface temperature was not measured; therefore, the thermal safety of these protocols could not be assessed, nor could it be inferred from our results. Although a low output power (100 mW) and a short irradiation time (90 s) were selected, in line with the manufacturer’s recommendations and previous reports to limit heat generation, this was not verified experimentally. Alfredo et al.^[12]^ showed that temperature increases at the external root surface depend directly on the laser parameters and must remain within safe limits to avoid damage to the periodontal tissues. The absence of temperature monitoring is therefore a limitation of the present study, and future investigations should include direct measurements of external root surface temperature to evaluate the thermal safety of these irradiation protocols.

 No significant difference was observed in CFU/mL reduction between NaOCl combined with the 808 nm laser (G3) and NaOCl alone (G1) (adjusted P = 0.34). In contrast, when the diode laser was used without NaOCl, it was significantly less effective than NaOCl alone: NaOCl alone (G1) produced a significantly greater reduction in CFU/mL than PDT alone (G4) (adjusted *P* = 0.04), followed by the 808 nm laser alone (G5) (adjusted *P* < 0.001).

 Although all disinfection protocols significantly reduced the bacterial load, none completely eliminated *E. faecalis*. From a clinical perspective, this finding reinforces the concept that complete root canal sterilization remains challenging, even when adjunctive technologies are incorporated into conventional irrigation protocols. Therefore, these approaches should be regarded as supplementary strategies to reduce bacterial burden rather than as substitutes for meticulous chemomechanical preparation and irrigation procedures.

 The persistence of residual bacteria may be explained, at least in part, by the anatomical complexity of the root canal system. Lateral canals, apical ramifications, isthmuses, and anatomical irregularities may serve as bacterial reservoirs that are difficult to access using both irrigants and laser irradiation. Ricucci and Siqueira^3^ emphasized that these anatomical complexities are important contributors to endodontic treatment failure because they may harbor persistent microorganisms and maintain communication with the periapical tissues.

 Another methodological aspect to consider is the microbiological sampling technique. Sterile paper points were selected because they represent a simple, standardized, and widely used method for recovering intracanal microorganisms in endodontic studies. However, this technique primarily samples bacteria in the main canal lumen and may not adequately recover microorganisms located deep within dentinal tubules or within complex biofilm structures. Considering the well-documented ability of *E. faecalis* to penetrate dentinal tubules, the actual persistence of bacteria may have been underestimated. Future studies should consider combining paper point sampling with dentin shaving techniques or other complementary methods to provide a more comprehensive assessment of residual intracanal bacterial survival.

 Although no a priori sample size calculation was performed, the post hoc power analysis based on the Kruskal-Wallis result showed a large observed effect size (ε^2^ = 0.728), indicating adequate statistical power to detect differences between the experimental protocols. Nevertheless, the use of 15 specimens per group may still limit the external validity and generalizability of the findings, particularly considering anatomical variability in root canals.

 Another limitation of this study is that no baseline microbiological assessment was conducted to quantitatively confirm equivalent bacterial loads across the experimental groups prior to treatment. Although all specimens underwent a standardized contamination protocol, including inoculation with a McFarland 0.5 suspension, a 14-day anaerobic incubation period, and five reinoculations to promote mature biofilm formation, some variability in bacterial colonization among specimens cannot be completely excluded. Future studies should incorporate baseline CFU measurements or positive contamination controls to confirm the homogeneity of biofilm formation before the application of disinfection protocols.

 Although single-rooted premolars with standardized preparation were selected in the present study, some degree of anatomical variability is inevitable in ex vivo investigations and may have influenced the antimicrobial outcomes observed among specimens. Consequently, future studies should investigate these protocols in more complex root canal anatomies and under clinical conditions to determine whether the superior antimicrobial performance observed with the NaOCl–PDT protocol can be reproduced in anatomically challenging clinical scenarios.

## Conclusion

 Under the tested ex vivo conditions, all active disinfection protocols showed lower *E. faecalis *CFU/mL counts than the saline control. Among them, the combination of 5.25% NaOCl with PDT (0.005% methylene blue and 660 nm irradiation) yielded the lowest CFU/mL counts and was significantly more effective than NaOCl alone (adjusted *P* = 0.01), followed by the laser protocols applied without NaOCl (adjusted *P* < 0.001). However, it was not significantly different from NaOCl combined with the 808 nm laser (adjusted *P* = 0.15); therefore, its lower counts represent a numerical ranking rather than a statistically confirmed superiority over all active protocols.

 When applied without NaOCl, the diode laser protocols were significantly less effective than NaOCl alone, indicating that laser systems should be regarded as adjunctive rather than standalone disinfection methods. No protocol achieved complete elimination of *E. faecalis*, reflecting the challenges imposed by the anatomical complexity of the root canal system.

 These findings should be interpreted within the limitations of an ex vivo design. Although residual NaOCl was neutralized with sodium thiosulfate before sampling to prevent carryover antimicrobial activity, baseline CFU counts were not obtained, microbiological sampling relied on paper points, and external root surface temperature was not monitored. Further studies using validated biofilm models, improved microbiological sampling methods, and clinical safety assessments are needed to confirm these results and to define the clinical role of laser-assisted protocols.

## Competing Interests

 The authors declare no conflict of interest.

## Ethical Approval

 This study was approved by the Ethics Committee of the University of the Hemispheres, Quito, Ecuador (protocol n. CEUHE25-47).
